# Comparing cestode infections and their consequences for host fitness in two sexual branchiopods: alien *Artemia franciscana* and native *A. salina* from syntopic-populations

**DOI:** 10.7717/peerj.1073

**Published:** 2015-07-02

**Authors:** Stella Redón, Francisco Amat, Marta I. Sánchez, Andy J. Green

**Affiliations:** 1 Instituto de Acuicultura de Torre de la Sal (IATS-CSIC), Ribera de Cabanes s/n, Castellón, Spain; 2 Department of Wetland Ecology, Estación Biológica de Doñana (EBD-CSIC), Américo Vespucio s/n, Sevilla, Spain

**Keywords:** Cestodes, Sexual *Artemia*, Syntopic populations, Invasive species, Host impact, Coevolution, Mediterranean salterns

## Abstract

The American brine shrimp *Artemia franciscana* is invasive in the Mediterranean region where it has displaced native species (the sexual *A. salina*, and the clonal *A. parthenogenetica*) from many salt pond complexes. *Artemia* populations are parasitized by numerous avian cestodes whose effects have been studied in native species. We present a study from the Ebro Delta salterns (NE Spain), in a salt pond where both *A. franciscana* and native *A. salina* populations coexist, providing a unique opportunity to compare the parasite loads of the two sexual species in syntopy. The native species had consistently higher infection parameters, largely because the dominant cestode in *A. salina* adults and juveniles (*Flamingolepis liguloides*) was much rarer in *A. franciscana*. The most abundant cestodes in the alien species were *Eurycestus avoceti* (in adults) and *Flamingolepis flamingo* (in juveniles). The abundance of *E. avoceti* and *F. liguloides* was higher in the *A. franciscana* population syntopic with *A. salina* than in a population sampled at the same time in another pond where the native brine shrimp was absent, possibly because the native shrimp provides a better reservoir for parasite circulation. Infection by cestodes caused red colouration in adult and juvenile *A. salina*, and also led to castration in a high proportion of adult females. Both these effects were significantly stronger in the native host than in *A. franciscana* with the same parasite loads. However, for the first time, significant castration effects (for *E. avoceti* and *F. liguloides*) and colour change (for six cestode species) were observed in infected *A. franciscana*. Avian cestodes are likely to help *A. franciscana* outcompete native species. At the same time, they are likely to reduce the production of *A. franciscana* cysts in areas where they are harvested commercially.

## Introduction

The American brine shrimp *Artemia franciscana* Kellog, 1906 is a key species for the aquaculture industry and a model organism for laboratory research (e.g., in toxicology, genetics or physiology). *A. franciscana* cysts, particularly originating from Great Salt Lake (USA), have been exported worldwide for aquaculture, the improvement of salt extraction in salt ponds and the pet trade market, facilitating the arrival and spread of *A. franciscana* outside its natural range (Amat et al., 2005; Ruebhart, Cock & Shaw, 2008; Vikas et al., 2012). In the Western Mediterranean, the introduction of this exotic species is provoking the extinction of native *A. salina* (Linnaeus, 1758) and *A. parthenogenetica* Bowen & Sterling, 1978 populations (Amat et al., 2005; Amat et al., 2007; Muñoz et al., 2014). The ability of *A. franciscana* to outcompete other *Artemia* species rapidly in the field may be largely explained by a higher reproductive rate that often allows it to eliminate native congeners within a few generations in the laboratory (Browne, 1980; Browne & Halanych, 1989; Amat et al., 2007). However, parasites can also influence biological invasions, depending on their relative impacts on native and alien species (Hatcher, Dick & Dunn, 2006; Prenter et al., 2004; Dunn et al., 2012).

*Artemia* spp. are intermediate hosts of avian cestodes that can have a major influence on their fitness. In the Mediterranean, native brine shrimps are parasitized by 12 species of avian tapeworms whose final hosts are flamingos, waders, grebes, ducks or gulls (Georgiev et al., 2005; Georgiev et al., 2007; Vasileva et al., 2009). To elucidate the role of parasites in an invasion it is important to study parasite infections in populations of native and alien host species co-ocurring in the same habitat (syntopic populations), but this is difficult because native *Artemia* have already disappeared from most sites where *A. franciscana* is detected. Comparisons of allopatric *Artemia* populations from the southern Iberian Peninsula suggest that *A. franciscana* populations have lower levels of infections by cestodes than the native sexual *A. salina* and the clonal *A. parthenogenetica* (Georgiev et al., 2007; Georgiev et al., 2014; Sánchez et al., 2013). In the present study, we take advantage of a unique opportunity to compare the parasitism of *A. franciscana* and *A. salina* in the only site where these two sexual species are known to coexist: the Ebro Delta salterns in north-east Spain.

Sánchez et al. (2012) compared parasitism in syntopic host populations of *A. franciscana* and *A. parthenogenetica* in southern France, and found lower cestode diversity and abundance in the invasive host. This could potentially be because the sexual invasive species can resist parasites better than the clonal native one, owing to the importance of genetic recombination in resisting parasites (Red Queen hypothesis: Van Valen, 1973; Moritz et al., 1991; Mee & Rowe, 2006). However, in the case of two sexual species, the relative effects of parasites may be more similar. Given the lack of preexisting data, comparing parasite impacts in two bisexual hosts (*A. franciscana* and *A. salina*) is of considerable interest in the context of the biological invasion.

Trophically transmitted parasites such as larval helminths with complex life cycles often induce changes in the physiology, behaviour or appearance of intermediate hosts (“host manipulation”), rendering them more vulnerable to predation and facilitating transmission to final hosts (Barber, Hoare & Krause, 2000; Moore, 2002; Poulin, 2006). In native Mediterranean *Artemia* populations, cestode infections are associated with a reddish colour, positive phototaxis, gigantism, longer life span, reduced fecundity and higher levels of lipids and glycogen (Thiéry, Robert & Gabrion, 1990; Amat et al., 1991; Robert & Gabrion, 1991; Sánchez et al., 2006; Sánchez, Georgiev & Green, 2007; Amarouayache, Derbal & Kara, 2009; Sánchez et al., 2009a). The pathogenic impact of endemic cestodes on the alien *A. franciscana* remains unclear, particularly when it co-occurs with native congeners. If *A. franciscana* is less affected by cestodes, this may help explain its invasion success by aiding it to outcompete native hosts.

In the present study, we compared the cestode infections between *A. franciscana* and *A. salina* in a salt pond where they coexisted. We tested the hypothesis that *A. franciscana* is less susceptible to native cestodes than *A. salina*, leading to a competitive advantage. Secondly, we compared the infections between this *A. franciscana* population that is coexisting with a native host (*A. franciscana*-syntopic population), and an *A. franciscana* population from a neighbouring pond where there are no congeners, in order to explore if there is a negative relationship between community diversity and disease risk (Schmidt & Ostfeld, 2001; Keesing, Holt & Ostfeld, 2006; Johnson & Thieltges, 2010). Thirdly, we analyzed the consequences of infection by different cestode species for colour change and reproductive activity in both *Artemia* species. We predicted fewer consequences for the alien host, owing to weaker host–parasite coevolution.

## Materials and Methods

### Study system and field samples

**Figure 1:**
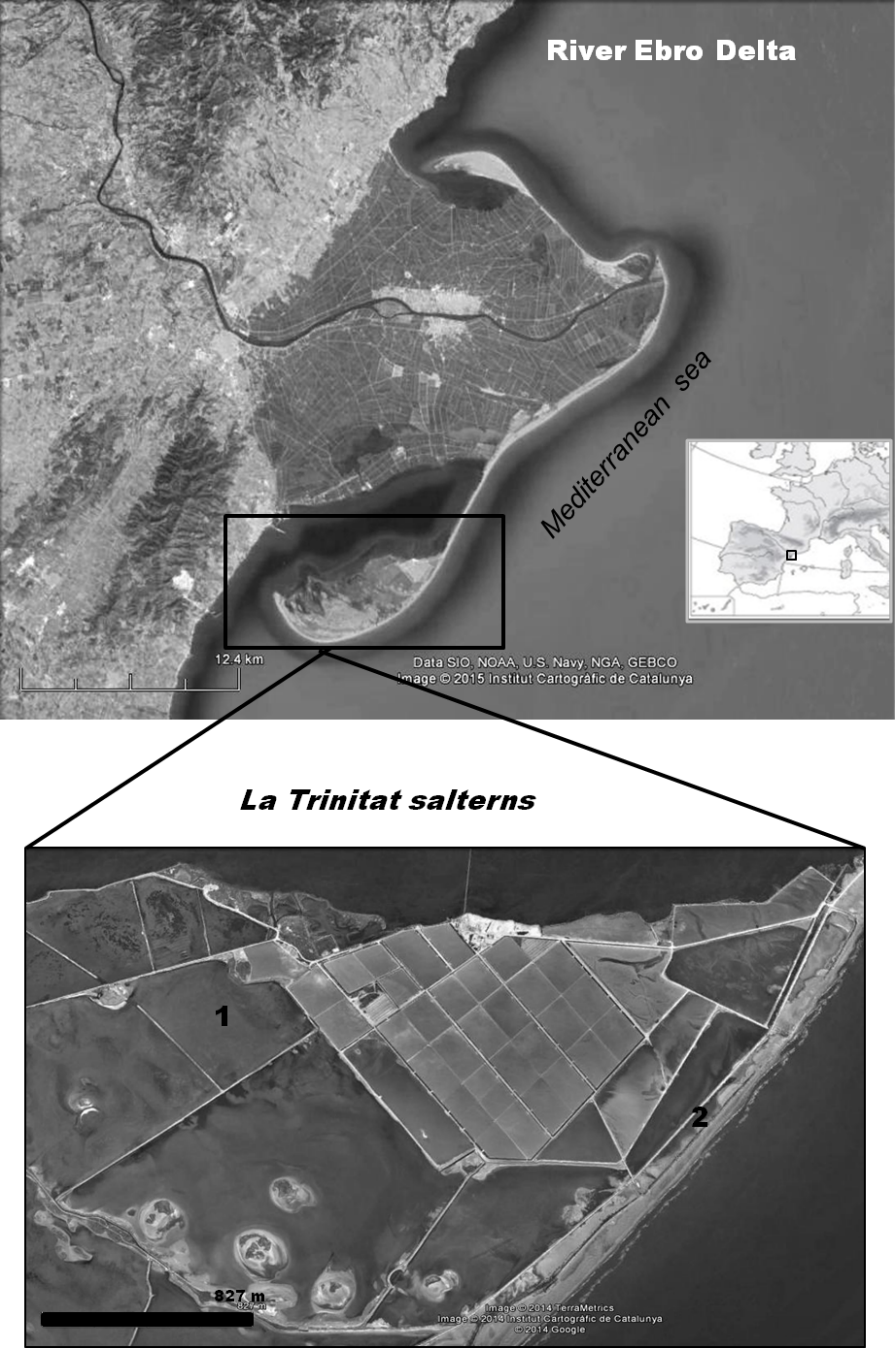
Geographical situation of the study area. Location of the Ebro Delta (Province of Tarragona, NE Spain) and map of the Ebro Delta salterns *La Trinitat* indicating the *Artemia* collection sites: (1) Pond 4, (2) Pond CX.

The Ebro Delta (Province of Tarragona, NE Spain) is the largest wetland area (320 km^2^) along the Mediterranean coast of Spain and is protected as a Natural Park, Ramsar site and an EU Special Protection Area for birds. *Artemia franciscana* was first detected in our study area (*La Trinitat* coastal salterns, 40°35′N, 00°41′E, Fig. 1) in 2007 (Amat et al., 2007). Previously, these salterns supported a tetraploid parthenogenetic population of *Artemia* (Amat et al., 1995), but this native taxon has not been recorded since.

Repeated sampling visits were carried out from 2007 to 2010 inclusive. The present study focuses on samples collected at the following two ponds during a 12 month period from January 2009 to January 2010, when the native *A. salina* was in coexistence with *A. franciscana*. Pond CX, a large pond situated between other salt ponds and the sea, isolated from the brine circulation system (Fig. 1), was the only pond in which *A. franciscana* coexisted with *A. salina*. Samples were collected monthly from January 2009 to January 2010 (salinity *S* range = 45–260 g/L, mean ± s.e. = 114.6 ± 18.35; temperature *T* range = 5–32 °C, mean ± s.e. = 18.1 ± 2.37). Pond 4 was sampled monthly from January to March 2009, then again in January 2010 (*S* range = 125–150 g/L, mean ± s.e. = 132.5 ± 5.95; *T* range = 8.5–12 °C, mean ± s.e. = 10.9 ± 0.83). In order to increase the sample size for infected shrimps, additional samples of *A. franciscana* collected on other dates and in other ponds (S Redón, AJ Green, BB Georgiev, GP Vasileva, F Amat, 2015, unpublished data) were used when considering the influence of cestodes on colouration and reproductive activity of this host species.

*Artemia* samples were collected from each pond with 160 µm and 500 µm mesh hand-nets and transported alive to the laboratory. Living *Artemia* individuals, anaesthetized with a few drops of distilled water saturated with chloroform, were examined under a stereomicroscope and juveniles and adults were separated. Juveniles are immature specimens with sexual segments (ovisac or hemipenis) that are not completely developed. Juveniles and adults were identified to species after Amat (1985), Hontoria & Amat (1992a) and Hontoria & Amat (1992b). The proportion of juveniles varied over time (Fig. S1) confirming that both species had multiple generations per year (Amat et al., 2007). Both juveniles and adults were sexed (Amat, 1985). Adult females with empty ovisac and no signs of functional ovaries were classified as castrated and those with embryos, naupliae, or cysts filling the ovisac, or ovulating (oocites moving along the ovaries or filling the oviducts) were classified as ovigerous (Fig. S3). The colour of adults and juveniles was assigned to three categories: dark-red, light-red and not-red (Fig. S2). This research was conducted under a permit from the Ebro Delta Natural Park office provided to FA.

### Parasite identification

Juvenile and adults were examined, while lightly anaesthetized, under a stereomicroscope for cestode cysticercoids, until a total of approximately 400 individual shrimps (when available) was reached, including juveniles and adults. After observations of the cysticercoids *in situ*, each infected specimen was prepared in a temporary glycerol mount and examined under a compound microscope. Identification was based on Georgiev et al. (2005) and Vasileva et al. (2009). More details of sampling protocols are provided by S Redón, AJ Green, BB Georgiev, GP Vasileva, F Amat (2015, unpublished data).

### Quantitative analysis of cestode infection and statistics

Several descriptors were applied to the cestode infections in *Artemia*. Prevalence (P%: proportion of infected individuals in the host population), abundance (MA: mean number of cysticercoids for the total number of shrimps examined) and intensity (MI: mean number of cysticercoids in infected shrimps) were calculated for the overall infection and for each parasite species (terminology following Bush et al., 1997). Also, we quantified species richness (SR: mean number of cestode species present in each *Artemia* individual) and the species richness in infected specimens (SRinfected).

(i) Host species: invader vs. native

To compare cestode infections between host species, we used monthly samples from pond CX in which a total of 2,150 shrimps were examined (1,252 *A. franciscana* and 898 *A. salina*). On a month by month basis, Mann–Whitney *U* tests were employed to compare the abundance of parasites, the infection intensity and SR between host species, separately for adults and juveniles. Differences in the prevalence of cestodes between host species were analyzed with Fisher Exact tests.

(ii) Influence of coexistence with *A. salina* on parasitism in *A. franciscana*

We compared the parasite infections in *A. franciscana* in a syntopic population (presence of *A. salina*, pond CX) with those in a single species population sampled simultaneously (pond 4). Using Fisher Exact and Mann–Whitney *U* tests, we compared the cestode infections in *A. franciscana* between ponds on the same sampling day during three months (January, February and March 2009), separately for adults and juveniles.

### Parasite castration effects

Taking advantage of all *A. franciscana* samples available (including those from other dates and ponds; S. Redón, AJ Green, BB Georgiev, GP Vasileva, F Amat, 2015, unpublished data), we compared the proportion of infected and uninfected females that were castrated. The castration effect of cestodes in *A. franciscana* was evaluated by Wilcoxon tests for paired samples, comparing the proportions of castrated shrimps within a given sample, thus avoiding any non-independence of observations of different individuals within the same sample. In contrast, Fisher Exact tests were applied for *A. salina*, because the small number of samples with this species obliged us to pool them and treat each individual shrimp as an independent observation. Since castration was never recorded in uninfected female *A. salina*, this pooling was unlikely to bias the observed levels of castration.

### Effects of parasites on colour

In order to assess the effects of parasites on the colour of *Artemia* individuals, we compared the proportion of red individuals (summing the “dark-red” and “light-red” categories, Fig. S2) between infected and non-infected specimens with Fisher Exact tests. The same test was employed to analyze colour-effects between host sexes and host species for both adults and juveniles. In addition, to test the influence of infection intensity on colour, Mann–Whitney *U* tests were applied to compare the cestode intensity between infected individuals that were red and those that were not. All statistical analyses were carried out using SPSS 15.0 for Windows (SPSS Inc., Chicago, IL, USA).

## Results

### Comparing parasite loads in *A. franciscana* and *A. salina* in syntopic populations

**Figure 2:**
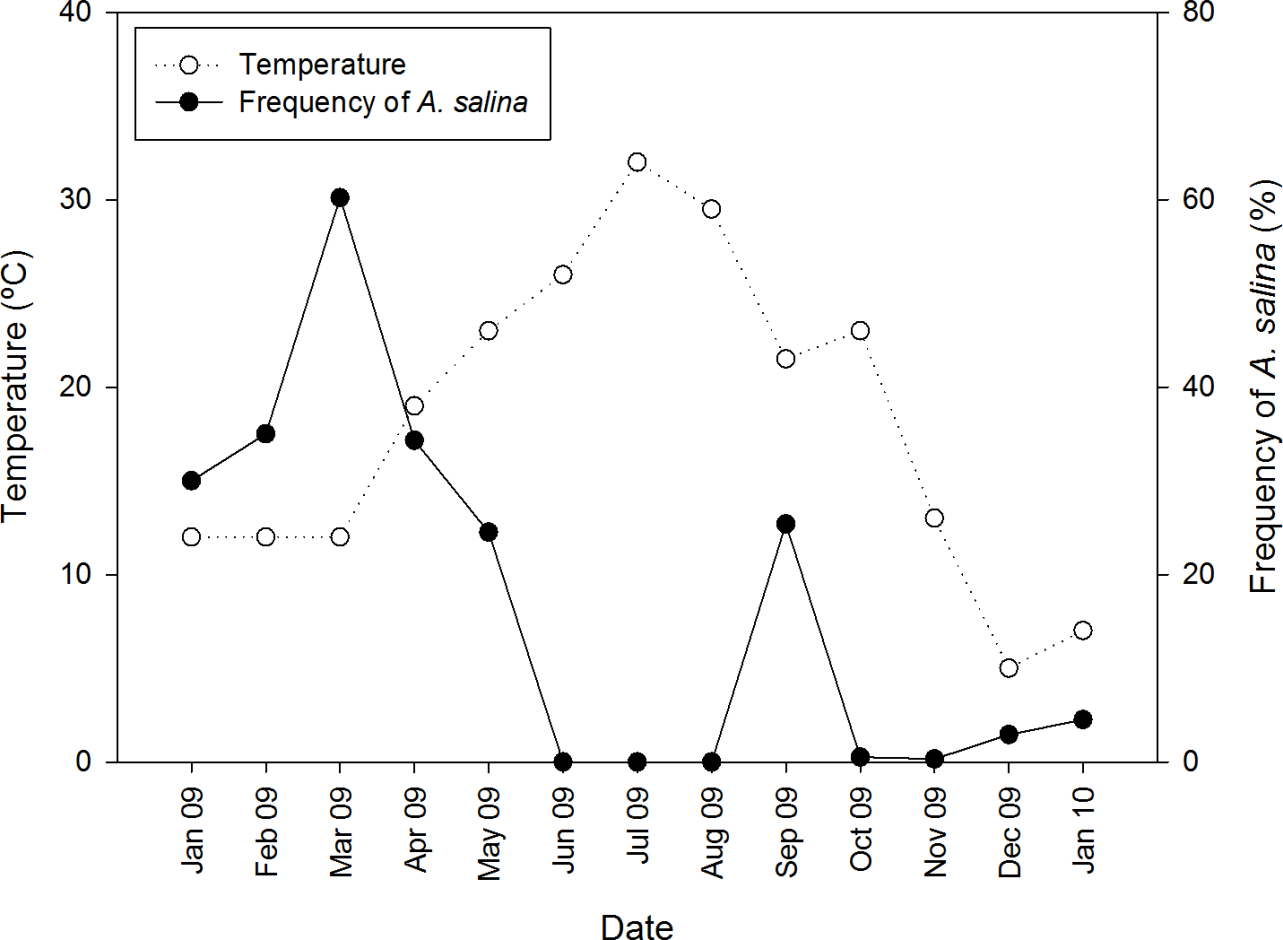
Seasonal variation of temperature and proportion of native *A. salina* in the brine shrimp community from pond CX throughout an annual cycle. The right vertical axis indicates the proportion of all *Artemia* present (whether adults or juveniles) that were *A. salina*. *A. franciscana* were present in all months.

In pond CX where both *Artemia* species coexisted, *A. salina* was detected only in colder months, from January to May 2009, and September 2009 to January 2010 (Fig. 2), whereas *A. franciscana* was present all year long. Nine cestode species were detected in *A. franciscana*, and seven of these in *A. salina* (Table 1). These included four species whose adults are parasitic in flamingos (*Flamingolepis liguloides*, *F. flamingo*, *Gynandrotaenia stammeri* and *Gynandrotaenia* sp.), three in shorebirds (*Anomotaenia tringae*, *A. microphallos* and *Eurycestus avoceti*), one in gulls (*Wardium stellorae*) and one in shelducks (*Fimbriarioides tadornae*).

**Table 1:** Comparative cestode infection in syntopic brine shrimp populations from pond CX (adults and juveniles). Only data for samples in which both *Artemia* species were recorded (*N* = 5) are presented.

		Host—adults		Host—juveniles	
**Cestode species**		*A. franciscana N* = 487	*A. salina N* = 381	*A. franciscana N* = 765	*A. salina N* = 517
*Flamingolepis liguloides* (FL)	P%	0.8	17.6	1.4	8.3
	MI	1.00 ± 0.00	1.25 ± 0.07	1.18 ± 0.18	1.21 ± 0.11
	MA	0.008 ± 0.004	0.221 ± 0.03	0.017 ± 0.01	0.101 ± 0.02
*Flamingolepis flamingo* (FF)	P%	2.3	1.3	2.2	1.2
	MI	1.09 ± 0.09	1.20 ± 0.20	1.00 ± 0.00	1.17 ± 0.17
	MA	0.025 ± 0.01	0.016 ± 0.01	0.022 ± 0.01	0.014 ± 0.01
*Wardium stellorae* (WS)	P%	1.4	1.0	0.0	0.0
	MI	1.14 ± 0.14	1.00 ± 0.00	0.00	0.00
	MA	0.016 ± 0.01	0.011 ± 0.01	0.00	0.00
*Fimbriarioides tadornae* (FT)	P%	1.4	0.0	0.4	0.4
	MI	1.00 ± 0.00	0.00	1.00 ± 0.00	1.00 ± 0.00
	MA	0.014 ± 0.01	0.00	0.004 ± 0.002	0.004 ± 0.003
*Eurycestus avoceti* (EA)	P%	8.6	3.9	0.7	0.2
	MI	1.02 ± 0.02	1.00 ± 0.00	1.00 ± 0.00	1.00
	MA	0.088 ± 0.01	0.039 ± 0.01	0.007 ± 0.003	0.002 ± 0.002
*Anomotaenia tringae* (AT)	P%	4.9	7.3	0.3	1.2
	MI	1.08 ± 0.08	1.14 ± 0.09	1.50 ± 0.50	1.17 ± 0.17
	MA	0.053 ± 0.01	0.084 ± 0.02	0.004 ± 0.003	0.014 ± 0.01
*Anomotaenia microphallos* (AM)	P%	0.8	0.0	0.0	0.0
	MI	1.00 ± 0.00	0.00	0.00	0.00
	MA	0.008 ± 0.00	0.00	0.00	0.00
*Gynandrotaenia stammeri* (GS)	P%	0.2	0.00	0.0	0.0
	MI	1.00	0.00	0.00	0.00
	MA	0.002 ± 0.002	0.00	0.00	0.00
*Gynandrotaenia* sp. (GSP)	P%	0.4	0.8	0.1	0.0
	MI	1.00 ± 0.00	1.00 ± 0.00	1.00	0.00
	MA	0.004 ± 0.003	0.008 ± 0.005	0.001 ± 0.001	0.00
**Total infection**	P%	19.1	24.9	3.9	9.5
	MI	1.15 ± 0.04	1.52 ± 0.08	1.40 ± 0.13	1.41 ± 0.14
	MA	0.220 ± 0.02	0.378 ± 0.04	0.055 ± 0.01	0.134 ± 0.02
*Species richness ± SE*		0.21 ± 0.02	0.32 ± 0.03	0.05 ± 0.01	0.11 ± 0.02
*SR infected*		1.10 ± 0.03	1.28 ± 0.06	1.30 ± 0.09	1.18 ± 0.06

**Notes.**

P% prevalence MI mean intensity MA mean abundance ±SE standard error SR infected species richness of infected individuals

#### Adult brine shrimps

**Figure 3:**
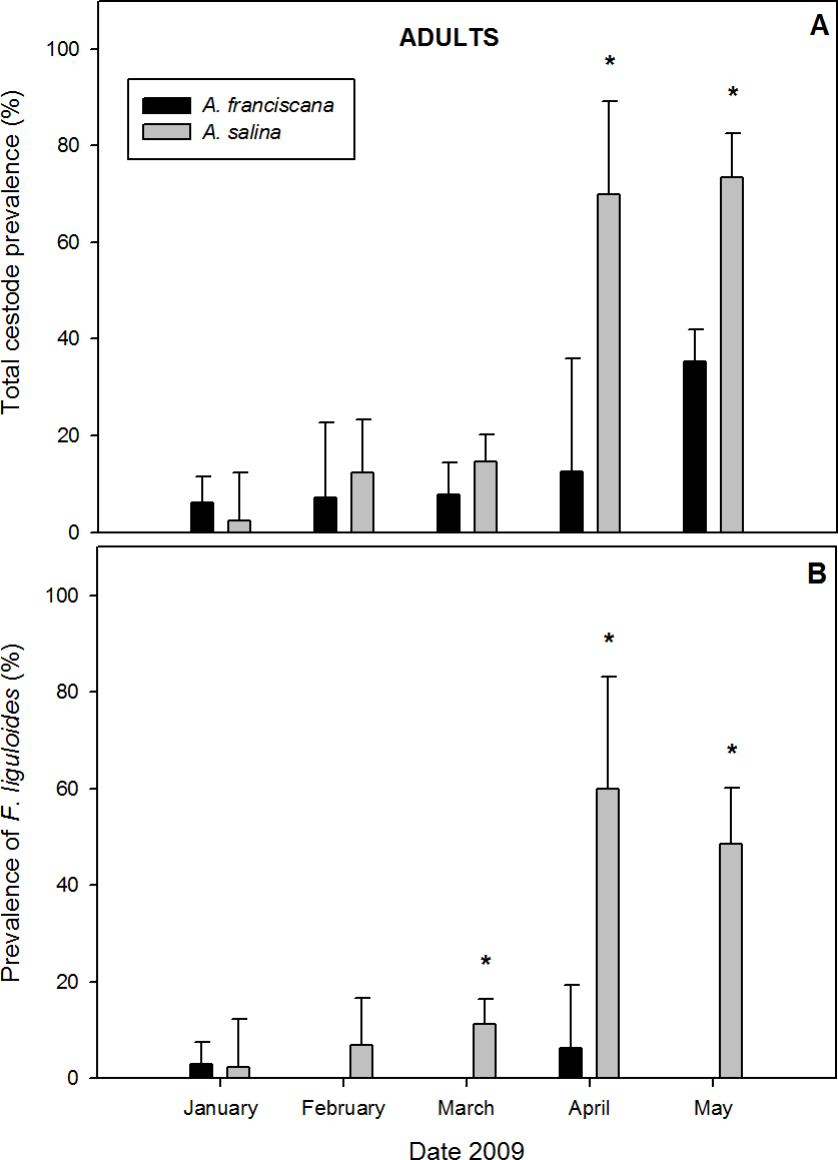
Comparative infection in adults of syntopic brine shrimp populations: *A. franciscana* and *A. salina* from pond CX, during months when they were co-existing. (A) Total cestode prevalence, (B) Prevalence of *Flamingolepis liguloides*. Bars show upper 95% confidence intervals. * significant differences at *p* < 0.05 according to Fisher Exact tests.

A total of 868 adults were examined in samples with both species present. Of these, 24.9% of *A. salina* and 19.1% of *A. franciscana* were parasitized by cysticercoids (Table 1). Cestodes were detected in all five months of co-existence (Fig. 3). For overall infection, *A. salina* had a higher prevalence than *A. franciscana* (Table 1), with statistically significant differences in April and May (Fig. 3A).

Mean total cestode abundance varied from 0.02 to 1.35 in *A. salina* and from 0.06 to 0.42 in *A. franciscana*, and was significantly higher in *A. salina* in April and May (Mann–Whitney *U* tests, *p* < 0.05). Total cestode intensity ranged from 1 to 1.84 in *A. salina* and 1 to 1.19 in *A. franciscana*, and was significantly higher in the former in May (*p* < 0.001). The relative abundance of cestode species varied between hosts. *F. liguloides* was the most prevalent and abundant parasite in *A. salina* (Table 1), and its prevalence and abundance were significantly lower in *A. franciscana* in March, April and May (Fig. 3B). In May, the prevalence and abundance of *A. tringae* were also significantly higher in *A. salina* (41.2% and 0.471 ± 0.08, respectively) than *A. franciscana* (11.6% and 0.126 ± 0.03). Except in January, *E. avoceti* was the most prevalent and abundant parasite in *A. franciscana* (Table 1). No significant differences in the intensity of individual cestode species were recorded (Table 1).

*A. salina* had a higher SR of cestodes (Table 1), being significantly higher in April (0.70 ± 0.15 for *A. salina vs*. 0.13 ± 0.09 for *A. franciscana*, *p* < 0.05) and May (1.12 ± 0.11 *vs*. 0.40 ± 0.04, *p* < 0.001). In May, the SR of infected specimens was also significantly higher for *A. salina* (1.52 ± 0.09 *vs*. 1.12 ± 0.04, *p* < 0.001).

#### Juvenile brine shrimps

**Figure 4:**
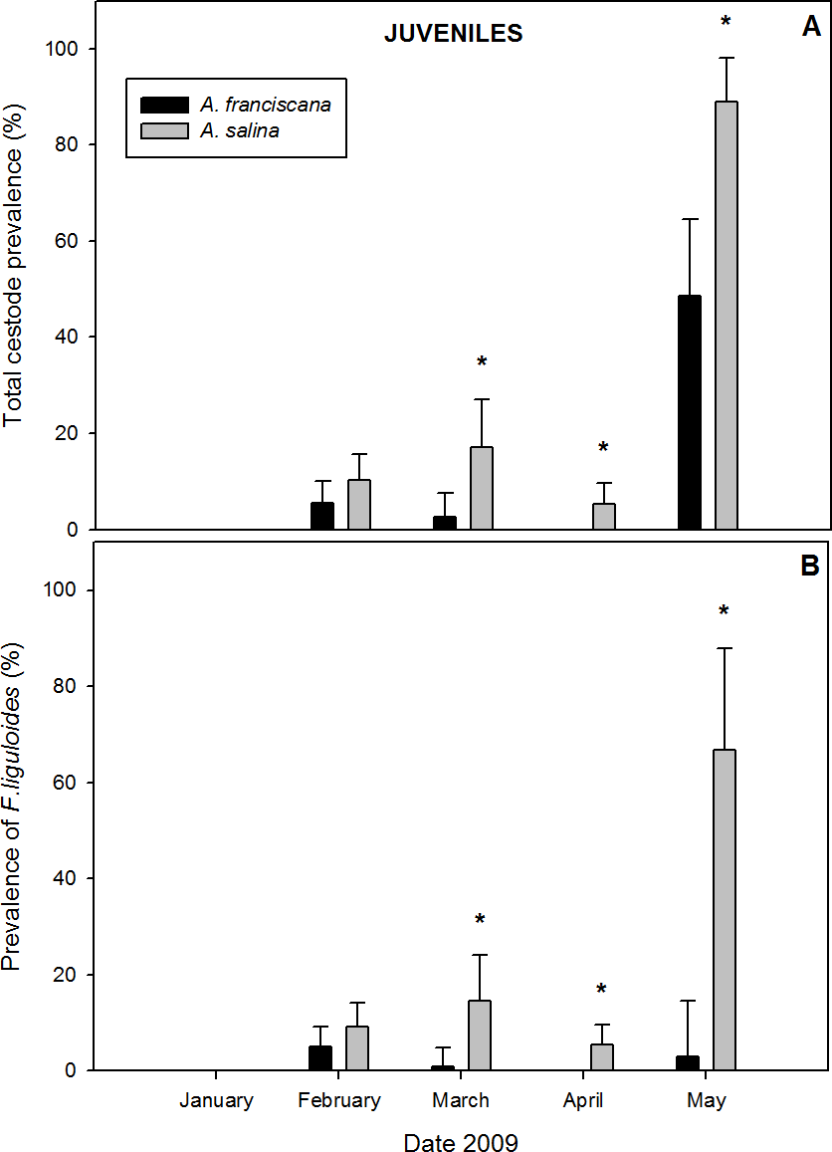
Comparative infection in juveniles of syntopic brine shrimp populations: *A. franciscana* and *A. salina* from pond CX, during months when they were co-existing. (A) Total cestode prevalence, (B) Prevalence of *Flamingolepis liguloides*. Bars show upper 95% confidence intervals. * significant differences at *p* < 0.05 according to Fisher Exact tests. No cestodes were recorded in January.

A total of 1,282 juveniles were examined in samples with both species present. Of these, 9.5% of *A. salina* and 3.9% of *A. franciscana* were parasitized by cysticercoids (Table 1). Cestodes were detected in all months of co-existence except January (Fig. 4). For the overall infection, prevalence and abundance were significantly higher in *A. salina* from March to May (Fig. 4A). No significant differences were detected in intensity between host species (Table 1).

Prevalence and abundance of *F. liguloides* were significantly higher in *A. salina* from March to May (Fig. 4B). Prevalence and abundance of *A. tringae* were also significantly higher in *A. salina* in May (66.7% *vs*. 5.7%, and 0.778 ± 0.22 *vs*. 0.086 ± 0.06). SR was significantly higher for *A. salina* in March (0.18 *vs.* 0.04; *p* = 0.001), April (0.05 *vs*. 0.00; *p* < 0.001) and May (1.56 *vs*. 0.63; *p* = 0.003). However, no significant differences were detected for SR of infected specimens.

### The influence of coexistence with native *A. salina* on parasitism in *A. franciscana*

For adult *A. franciscana*, higher total prevalence and abundance of cestodes were recorded in each of three months in pond CX where it was coexisting with *A. salina* (AF-syntopic) than in pond 4 where it was the only *Artemia* species present (AF-single), with statistically significant differences in March (Table 2). Prevalence was significantly higher in AF-syntopic for *F. liguloides* and *E. avoceti* in January and March, respectively (Table 2). SR was significantly higher in AF-syntopic in March (Table 2). No significant differences were detected for infection intensity.

**Table 2:** Comparative cestode infection in adult *A. franciscana* from single and syntopic populations. Parasite infections from a population without co-ocurrence of congeners (pond 4, AF-single) and one in co-existence with *A. salina* (pond CX, AF-syntopic) from January to March 2009. The proportion of adult *Artemia* that were *A. franciscana* is shown in parentheses. Fisher Exact tests were used to compare infection rates between ponds in a given month. Significantly higher values are shown in bold.

		January 2009			February 2009			March 2009		
**Cestode species**		AF-single *N* = 963, *S* = 125 g/l	AF-syntopic (75.9%) *N* = 132, *S* = 56 g/l	*p* value	AF-single *N* = 244, *S* = 130 g/l	AF-syntopic (32.9%) *N* = 28, *S* = 65 g/l	*p* value	AF-single *N* = 186, *S* = 150 g/l	AF-syntopic (33.8%) *N* = 104, *S* = 70 g/l	*p* value
FL	P%	0.3	**3.0** ^*^	0.005	0.8	0.0	1.000	0.0	0.0	–
	MI	1.00 ± 0.00	1.00 ± 0.00	1.000	1.00 ± 0.00	0.00	–	0.00	0.00	–
	MA	0.0031 ± 0.00	**0.0303** ± 0.01^**^	0.000	0.0082 ± 0.01	0.00	0.631	0.00	0.00	1.000
FF	P%	0.7	2.3	0.011	2.5	0.0	1.000	0.0	1.0	0.359
	MI	1.14 ± 0.14	1.00 ± 0.00	0.833	1.00 ± 0.00	0.00	–	0.00	1.00	–
	MA	0.0083 ± 0.00	0.0303 ± 0.01	0.081	0.0246 ± 0.01	0.00	0.402	0.00	0.0096 ± 0.01	0.181
WS	P%	0.0	0.0	–	0.0	0.0	–	0.5	0.0	1.000
	MI	0.00	0.00	–	0.00	0.00	–	1.00	0.00	–
	MA	0.00	0.00	1.000	0.00	0.00	1.000	0.0054 ± 0.01	0.00	0.455
FT	P%	0.2	0.0	1.000	0.8	3.6	0.279	0.0	0.0	–
	MI	1.00 ± 0.00	0.00	–	1.00 ± 0.00	1.00	1.000	0.00	0.00	–
	MA	0.0021 ± 0.00	0.00	0.600	0.0082 ± 0.01	0.0357 ± 0.04	0.187	0.00	0.00	1.000
EA	P%	1.7	0.8	0.710	0.8	3.6	0.279	0.0	**4.8** ^*^	0.006
	MI	1.00 ± 0.00	1.00	1.000	1.00 ± 0.00	1.00	1.000	0.00	1.00 ± 0.00	–
	MA	0.0166 ± 0.00	0.0076 ± 0.01	0.431	0.0082 ± 0.01	0.0357 ± 0.04	0.187	0.00	**0.0481** ± 0.02^*^	0.003
GS	P%	0.3	0.0	1.000	0.4	0.0	1.000	0.0	0.0	–
	MI	1.00	0.00	–	1.00 ± 0.00	0.00	–	0.00	0.00	–
	MA	0.0041 ± 0.00	0.00	0.521	0.0031 ± 0.00	0.00	0.735	000	0.00	1.000
GSP	P%	0.5	0.0	1.000	0.0	0.0	–	0.0	1.9	0.128
	MI	1.00 ± 0.00	0.00	–	0.00	0.00	–	0.00	1.00 ± 0.00	–
	MA	0.0052 ± 0.00	0.00	0.407	0.00	0.00	1.000	0.00	0.0192 ± 0.01	0.058
**Total infection**										
	P%	3.7	6.1	0.232	5.3	7.1	0.658	0.5	**7.7** ^*^	0.001
	MI	1.03 ± 0.03	1.00 ± 0.00	0.917	1.00 ± 0.00	1.00 ± 0.00	1.000	1.00	1.00 ± 0.00	1.000
	MA	0.0384 ± 0.01	0.0606 ± 0.02	0.204	0.0533 ± 0.01	0.0714 ± 0.05	0.691	0.0054 ± 0.01	**0.0865** ± 0.03^*^	0.001
*Species Richness*		0.04 ± 0.01	0.06 ± 0.02	0.203	0.05 ± 0.01	0.07 ± 0.05	0.691	0.01 ± 0.01	**0.08 ± 0.03** ^*^	0.001
*SR infected*		1.00 ± 0.00	1.00 ± 0.00	1.000	1.00 ± 0.00	1.00 ± 0.00	1.000	1.00	1.00 ± 0.00	1.000

**Notes.**

*N* total number of specimens examined *S* salinity

Other abbreviations are explained in Table 1.

* *p* < 0.05.

** *p* < 0.001.

– Test not possible owing to lack of data.

Amongst juvenile *A. franciscana*, total prevalence, abundance and species richness were significantly higher in AF-syntopic in March (as for adults), but significantly lower in AF-syntopic in January (when no infected juveniles of either *Artemia* species were recorded in the syntopic population). The abundance of *F. flamingo* in March was also significantly higher for AF-syntopic (Table 3).

**Table 3:** Comparative cestode infection in juvenile *A. franciscana* from single and syntopic populations. Parasite infections from a population without co-occurrence of congeners (pond 4, AF-single) and one in co-existence with *A. salina* (pond CX, AF-mixed) from January to March 2009. Proportion of juvenile *Artemia* that were *A. franciscana* is shown in parentheses. Fisher Exact tests were used to compare infection rates between ponds in a given month. Significantly higher values are shown in bold.

		January 2009			February 2009			March 2009		
**Cestode species**		AF-single *N* = 168	AF-syntopic (65.7%) *N* = 134	*p* value	AF-single *N* = 67	AF-syntopic (50.4%) *N* = 179	*p* value	AF-single *N* = 248	AF-syntopic (59.4%) *N* = 111	*p* value
FL	P%	1.2	0.0	0.505	0.0	5.0	0.119	0.0	0.9	0.309
	MI	1.00 ± 0.00	0.00	–	0.00	1.00 ± 0.00	–	0.00	1.00	–
	MA	0.0119 ± 0.01	0.00	0.206	0.00	0.0503 ± 0.02	0.062	0.00	0.0090 ± 0.01	0.135
FF	P%	1.8	0.0	0.257	1.5	1.7	1.000	0.0	1.8	0.095
	MI	1.33 ± 0.33	0.00	–	1.00	1.00 ± 0.00	1.000	0.00	1.00 ± 0.00	–
	MA	0.0238 ± 0.01	0.00	0.121	0.0149 ± 0.01	0.0168 ± 0.01	0.919	0.00	**0.0181** ± 0.01^*^	0.034
EA	P%	1.8	0.0	0.257	0.0	0.0	–	0	0.9	0.309
	MI	1.00 ± 0.00	0.00	–	0.00	0.00	–	0.00	1.00	–
	MA	0.0179 ± 0.01	0.00	0.121	0.00	0.00	1.000	0.00	0.0090 ± 0.01	0.135
GS	P%	0.6	0.0	1.000	0.0	0.0	–	0.0	0.0	–
	MI	1.00	0.00	–	0.00	0.00	–	0.00	0.00	–
	MA	0.0060 ± 0.01	0.00	0.372	0.00	0.00	1.00	000	0.00	1.000
GSP	P%	0.6	0.0	1.000	0.0	0.6	1.00	0.0	0.0	–
	MI	1.00	0.00	–	0.00	1.00	–	0.00	0.00	–
	MA	0.0060 ± 0.01	0.00	0.372	0.00	0.0056 ± 0.01	0.541	0.00	0.00	1.000
**Total infection**										
	P%	**6.0** ^*^	0.0	0.003	1.5	5.6	0.298	0.0	**2.7** ^*^	0.029
	MI	1.10 ± 0.10	0.00	–	1.00	1.30 ± 0.15	0.727	0.00	1.33 ± 0.33	–
	MA	**0.0655** ± 0.02^*^	0.00	0.004	0.0149 ± 0.01	0.0726 ± 0.02	0.165	0.00	**0.0360** ± 0.02^*^	0.009
*Species richness*		**0.06** ± 0.02^*^	0.00	0.004	0.01 ± 0.01	0.07 ± 0.02	0.165	0.00	**0.04** ± 0.02^*^	0.009
*SR infected*		1.00 ± 0.00		–	1.00	1.30 ± 0.15			1.33 ± 0.36	–

**Notes.**

*N* total number of specimens examined

Other abbreviations are explained in Table 1.

* *p* < 0.05.

– Test not possible owing to lack of data.

### Influence of parasites on host colouration

**Figure 5:**
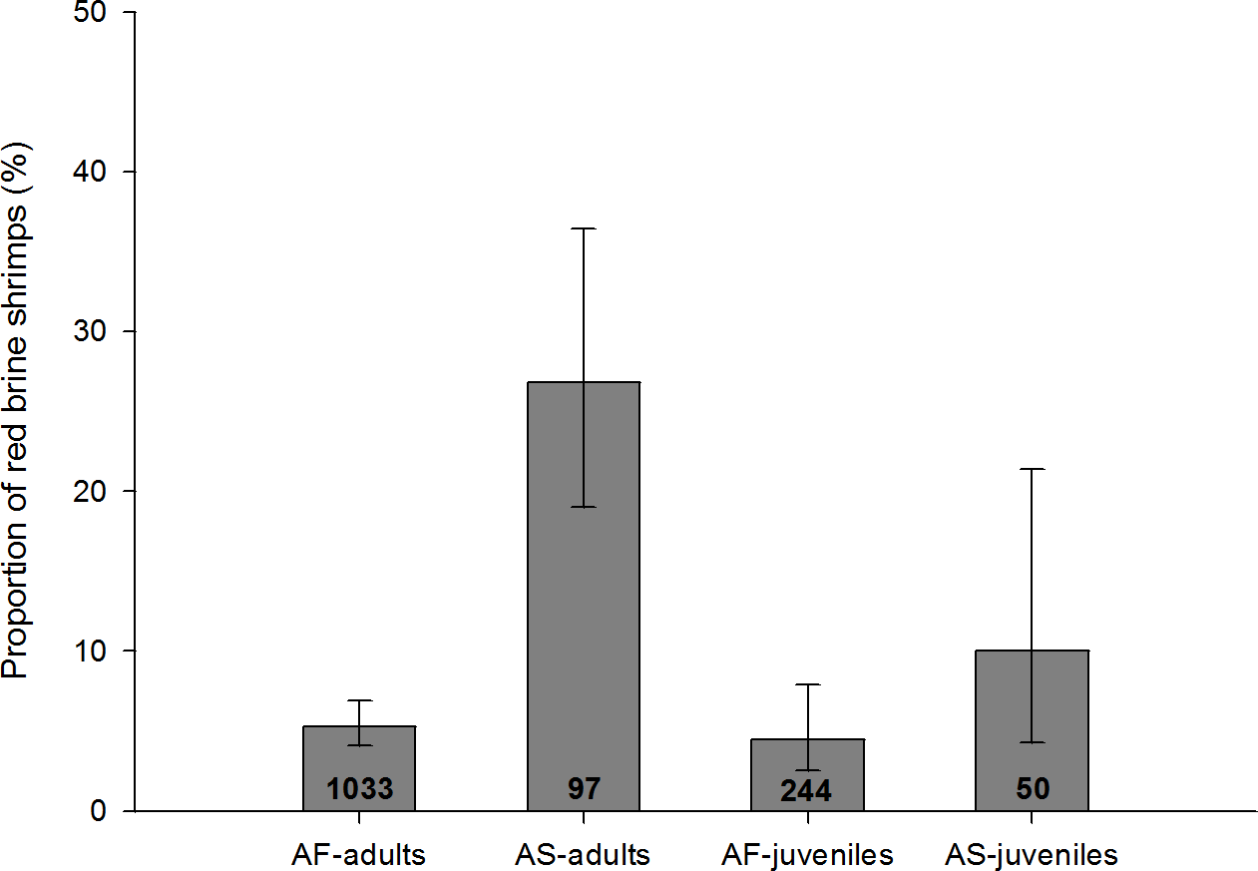
Proportion of infected adults and juveniles with red colouration for *A. salina* (AS) and *A. franciscana* (AF). Bars show 95% confidence intervals. The total number of infected specimens in each group is shown within the columns. Red colouration was not recorded in uninfected *A. salina*, and was very rare in uninfected *A. franciscana* (absent in juveniles, 0.02% in adults). For all four categories the proportion of red shrimps is significantly higher in infected individuals (Fisher Exact tests, *p* < 0.001).

Red colouration was strongly associated with presence of cestodes in both *Artemia* species, and in both adults and juveniles (Fig. 5 and Table 4). Among infected adults, red colouration was significantly more frequent in *A. salina* than *A. franciscana* (Table 4). There were no differences between sexes for either host species in the probability of redness when infected.

**Table 4:** Effects of infection status on colouration of *Artemia*. Data are presented for adults and juveniles of *A. franciscana* and *A. salina* from Ebro Delta salterns. The total number of individuals examined (including males and females) is given in parentheses. See Table 1 for abbreviations of parasite species. Different superscript letters denote significant differences between *Artemia* species of the same age group in the proportion of red individuals (Fisher Exact test, *p* < 0.05). Asterisks indicate a significant difference in the proportion of red individuals with the uninfected group.

	Adults		Juveniles	
	***A. franciscana***	***A. salina***	***A. franciscana***	***A. salina***
	% Red	% Red	% Red	% Red
Uninfected individuals	0.02% (8,260)	0% (325)	0% (8,658)	0% (507)
Infected individuals	5.3%^**^^a^ (1,033)	26.8%^**^^b^ (97)	4.5%^**^ (244)	10%^**^ (50)
**Infection status**				
Single infections (1 species)—Total	4.2%^**^^a^ (931)	12.2%^**^^b^ (74)	4.1%^**^ (221)	0% (41)
Infected only by FL	2.3%^*^ (86)	0% (47)	0% (32)	0% (35)
Infected only by FF	1.3%^*^ (79)	0% (2)	5.3%^**^ (114)^a^	0% (5)
Infected only by WS	0% (26)	0% (3)	0% (2)	–
Infected only by FT	0% (12)	–	0% (7)	–
Infected only by EA	4.2%^**^ (620)^a^	12.5%^*^ (8)^a^	2.3%^*^ (42)^a^	0% (1)
Infected only by AT	13.6%^**^^a^ (22)^a^	72.7%^**^^b^ (11)^a^	–	–
Infected only by AM	18.2%^**^ (33)^a^	–	66.7%^**^ (3)^a^	–
Infected only by GS	2.3%^*^ (43)^a^	–	0% (9)	–
Infected only by GSP	0% (7)	0% (3)	0% (2)	–
Multiple infections (≥2 species)	15.7%^**^^a^ (102)	73.9%^**^^b^ (23)	8.7%^**^^a^ (23)	55.6%^**^^b^ (9)

**Notes.**

* Fisher Exact test, *p* < 0.05.

** Fisher Exact test, *p* < 0.001.

a indicates cases where a single cysticercoid of that species was enough to cause a significant increase in the probability of redness (Fisher Exact test, *p* < 0.05).

For both *Artemia* species, there was a positive relationship between infection level (in terms of species richness and/or intensity of infection) and the likelihood of red colour.

**Figure 6:**
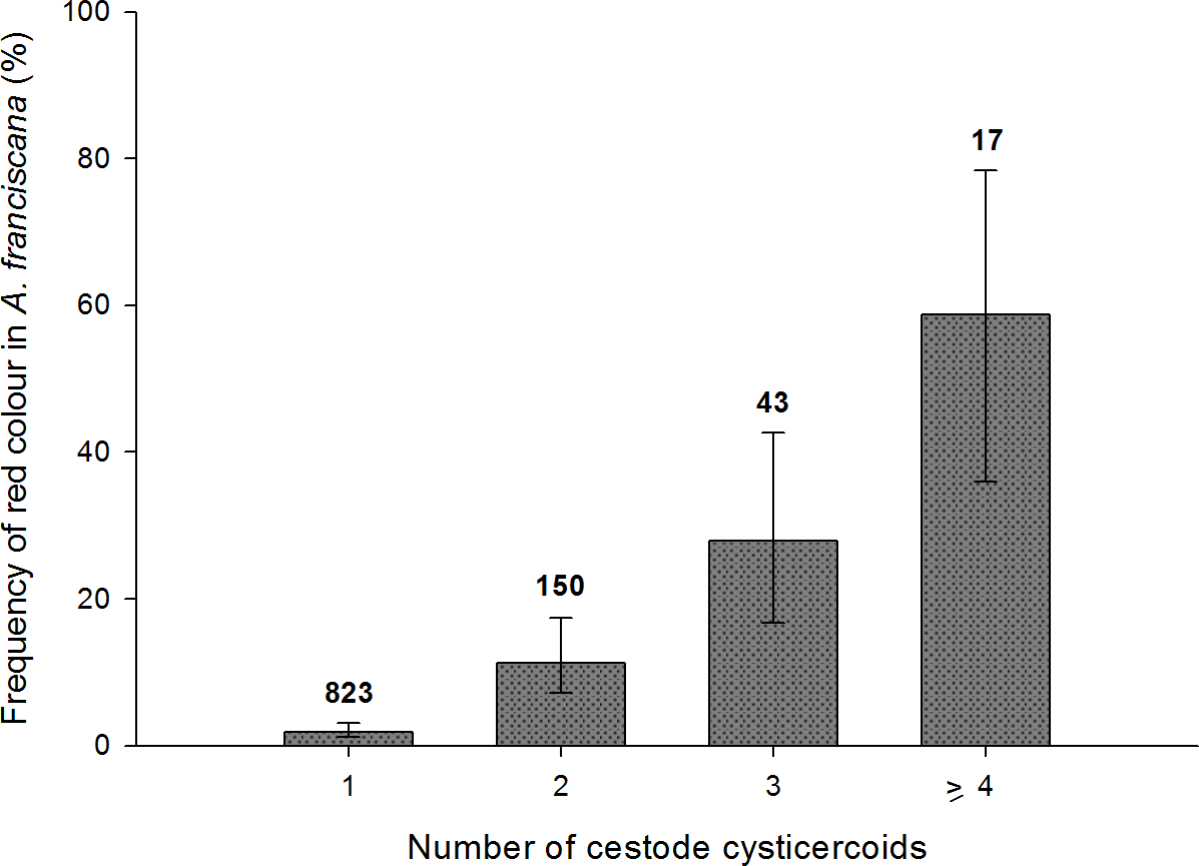
Colour pattern in relation to intensity of infection in adult *A. franciscana*. Bars show 95% confidence intervals. The total number of infected specimens in each group is indicated.

For infected adults, the total number of cysticercoids was higher in red individuals both for *A. franciscana* (mean ± s.e. = 2.4 ± 0.18 when red, 1.23 ± 0.02 when not; Mann–Whitney *U* test, *U* = 11,280, *p* < 0.001) and *A. salina* (2.08 ± 0.20 when red, 1.31 ± 0.07 when not; *U* = 487, *p* < 0.001). A similar result was obtained for infected juvenile *A. salina* (3.8 ± 0.58 when red, 1.16 ± 0.06 when not; *U* = 4, *p* < 0.001). Owing to the larger sample size, the positive correlation between infection intensity and probability of red colouration was particularly clear in *A. franciscana* adults (Fig. 6), for which the same pattern was apparent for the dominant cestode species, *E. avoceti* (results not shown).

Where only a single species of cestode was present (single infections), there was a significant redness effect in the case of six species for *A. franciscana* adults, three species for *A. franciscana* juveniles and two species for *A. salina* adults (Table 4). In most of these cases, the presence of a single cysticercoid was enough to cause a significant effect (Table 4). For *A. salina*, no colour-effects were observed for single infections of the most abundant cestode, *F. liguloides* (Table 4).

Simple infection by a single *A. tringae* cysticercoid was significantly more likely to cause redness in adult *A. salina* (*N* = 10, of which 70% were red) than in adult *A. franciscana* (*N* = 21, with 9.5% red) (Fisher Exact test, *p* = 0.001). No significant differences between host species in colour-effects were detected for other cestode species, although sample sizes for *A. salina* were small (Table 4).

### Cestodes and host castration

**Figure 7:**
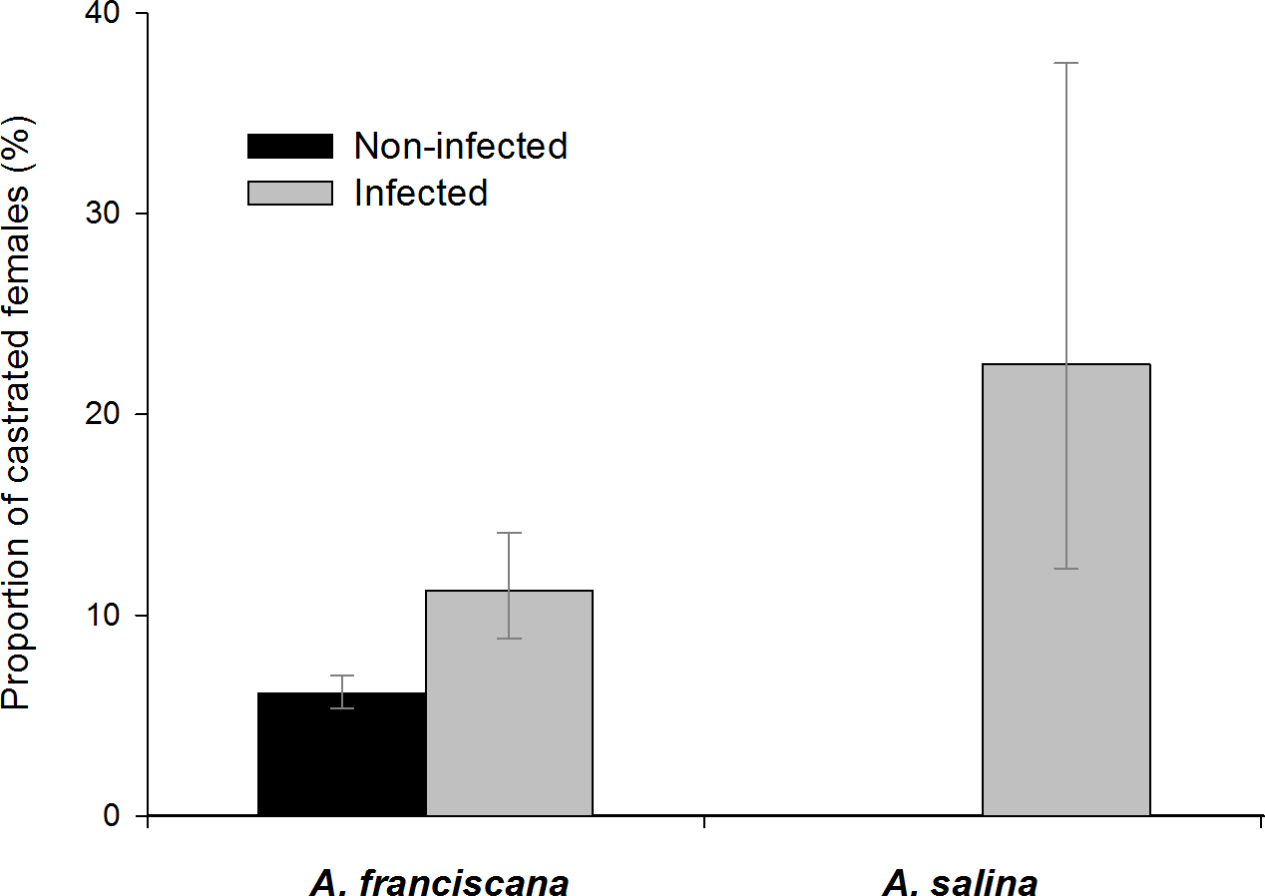
Castration effects in adult female *A. franciscana* and *A. salina*. Bars show 95% confidence intervals.

In *A. franciscana* adults, a higher proportion of infected females were castrated (i.e., with empty ovisac and no signs of functional ovaries, Fig. S3) than females which were not infected (Fig. 7 and Table 5).

**Table 5:** Castration effects associated with cestode infection in *Artemia*. Data are presented for adult females of the alien *A. franciscana* and native *A. salina*. Asterisks indicate significant differences in the proportion of castrated females with uninfected females of the same *Artemia* species. Wilcoxon Signed Rank tests were conducted to compare the proportions of uninfected and infected *A. franciscana* that were castrated in different samples, for total infections and for those infected only by EA. Fisher Exact tests were conducted for other taxa after pooling samples (see methods for details).

	*A. franciscana*		*A. salina*	
	*N* total	% castration	*N* total	% castration
Uninfected females	3,267	6.1%	111	0%
Infected females—Total	553	11.2%^*^	40	22.5%^**^
Single infections (1 species)—Total	496	8.5%^*^	29	20.7%^**^
Infected only by FL	32	25%^*^	14	35.7%^**^
Infected only by FF	28	7.1%	1	0%
Infected only by WS	14	0%	1	0%
Infected only by EA	362	7.5%^*^	5	0%
Infected only by AT	9	0%	5	20%^*^
Infected only by AM	22	4.5%	0	–
Infected only by GS	21	14.3%	0	–
Infected only by GSP	1	100%	3	0%

**Notes.**

*N* number of females examined

See Table 1 for abbreviations of parasite species.

* *p* < 0.05.

** *p* < 0.001.

Among infected, castrated female *A. franciscana*, 42 (68%) were infected by only one parasite species (i.e., single infections, Table 5), and of these most were infected with *E. avoceti* (64.3%) or *F. liguloides* (19%). Castration rates in females infected only by *E. avoceti* or only by *F. liguloides* were significantly higher than those in uninfected females (Table 5). For *E. avoceti*, the probability of castration clearly increased with the intensity of infection, and 35.7% of females with ≥3 cysticercoids were castrated (*p* = 0.001). Unlike *E. avoceti*, infection with a single *F. liguloides* cysticercoid had a significant castration effect (*p* = 0.004). Of female *A. franciscana* infected by more than one cestode species (co-infections) (*N* = 57), 35.1% were castrated. Among these castrated females (*N* = 20), *E. avoceti* was present in 18 individuals and *F. flamingo* in seven.

For *A. salina*, castration was only recorded in infected females (Fig. 7 and Table 5). Castration effects were significant for single infections by *F. liguloides* or by *A. tringae* (Table 5). If we include females with small broods (<10 eggs in the ovisac) and no oocytes migrating in the ovaries, the proportion of female *A. salina* with limited fecundity when infected with *F. liguloides* increases from 35.7% to 57.1%.

## Discussion

We present a unique study comparing the cestode infections and their consequences for two closely related branchiopod crustaceans. The native *A. salina* generally had more cestodes than the alien *A. franciscana*. When coexisting with the native, the alien had higher rates of infection than when alone. Although the cestodes were shown to have important effects on the fecundity and colour of both host species, these effects were stronger in the native species.

### Comparing infections in syntopic *A. franciscana* and *A. salina*: how important is co-evolution?

Comparative studies of parasite infections in native and alien host species are essential to understand the role of parasites in biological invasions (Dunn, 2009; Kelly et al., 2009; Dunn et al., 2012). The present work sheds light on the role of endemic cestodes in competitive interactions between native and alien sexual *Artemia*. Cestode parasitism differed clearly between invasive and native brine shrimps under the same environmental conditions and at the same time. Cestode species richness and the prevalence of several species were lower in *A. franciscana*, and the difference was especially striking for *F. liguloides* (Figs. 3 and 4). These results are consistent with studies of populations in the southern Iberian Peninsula which have found *A. franciscana* to have low infection rates throughout the annual cycle. In summer, Georgiev et al. (2007) found lower levels of cestode infections in three *A. franciscana* populations than in an allopatric *A. salina* population. Studies throughout the annual cycle of *A. franciscana* in Cadiz Bay and of *A. salina* in Almería also found that the invasive species had fewer infections (Sánchez et al., 2013; Georgiev et al., 2014). Our results for syntopic populations indicate that these earlier results are not explained by other differences between these allopatric populations (e.g., in environmental conditions, or in the density of birds). A previous study in syntopic populations in Aigües-Mortes in France showed that *A. franciscana* had fewer cestodes than native, clonal *A. parthenogenetica* (Sánchez et al., 2012), although this might be related to the advantages of sex in resisting parasites. In any case, the present study provides strong evidence that the introduced *A. franciscana* is a less susceptible host for cestodes than the native sexual *A. salina*.

The lower burden of parasites in introduced hosts suggests *A. franciscana* is resistant to several native cestode species such as *F. liguloides*. Different capacities to infect the new host may be the result of varying host–parasite co-evolutionary history. The lower infection of *F. liguloides* in *A. franciscana* may be explained by a strong immune response of the host against this parasite, given the restricted distribution of flamingos in North America which barely overlaps with the natural range of *A. franciscana* (Muñoz et al., 2013). There are no flamingos in the western USA, from where *A. franciscana* has been exported around the world. Observations of dead cysticercoids of *F. liguloides* in *A. franciscana* support a strong immune response in this host (Georgiev et al., 2014). In contrast, the high prevalence of *E. avoceti* recorded in *A. franciscana* in our study may reflect a shared coevolutionary history, since *E. avoceti* occurs in shorebirds in North America (Clark, 1954). *A. franciscana* does not appear to be especially resistant to cestodes in its native range, since the total prevalence of cestodes in Great Salt Lake, USA was much higher than in the Ebro Delta and other parts of the invasive range (Redón et al., 2015). Our results are consistent with previous studies that have found parasites of native hosts to have no or a limited capacity to spread to non-indigenous congeners (Dunn & Dick, 1998; Torchin, Byers & Todd, 2005; Genner, Michel & Todd, 2008).

### How does coexistence of both species influence infection parameters in *Artemia franciscana*?

When a non-indigenous species arrives in a new range, it can affect native communities through different processes: “spillover” of introduced parasites, “spillback” of native parasites, and “dilution” of parasitism (Kelly et al., 2009). *A. franciscana* was introduced into Europe as imported cysts (dormant eggs) free of native cestode parasites, so “spillover” is not expected in this system. The low levels of parasitism recorded in *A. franciscana* indicate that it is a poor or incompetent reservoir for native cestodes, making “spillback” to native *Artemia* unlikely. We might expect a parasite dilution effect of the invader to the benefit of the native host which should be subjected to lower disease transmission when mixed with incompetent, alien hosts (Keesing, Holt & Ostfeld, 2006; Hall et al., 2009; Johnson & Thieltges, 2010). A reduced infection rate by native trematodes was demonstrated experimentally in native freshwater snails *Potamopyrgus antipodarum* exposed together with the invader *Lymnaea stagnalis* (Kopp & Jokela, 2007). Given the strong effects of cestodes on *A. salina*, such a “dilution effect” could increase the possibility of local persistence of *A. salina* in the presence of the invader (Hatcher, Dick & Dunn, 2006).

Because our study area did not include ponds holding only *A. salina*, we cannot reliably assess the strength of any dilution effect on this species. However, the levels of cestode prevalence we recorded in the presence of *A. franciscana* were not unusually low compared to studies of *A. salina* elsewhere in the absence of the invader (Table S1). Likewise, Sánchez et al. (2012) found no evidence of a dilution effect on *A. parthenogenetica* when coexisting with *A. franciscana*, since the total prevalence in *A. parthenogenetica* was high (c. 70%).

Our study allowed a good test of the “dilution effect” in the other direction, i.e., whether the native host reduced parasitism in the alien host. We found the opposite (an “amplification effect”), with higher infection in *A. franciscana* when coexisting with *A. salina*. Indeed, the temporal dynamics of infection in *A. franciscana* in pond CX (with both *Artemia* species) seem to depend on the proportion of native hosts in the *Artemia* population. Thus, total prevalence increased from January to May 2009 (coinciding with the abundance of *A. salina*), then declined from June to December when *A. franciscana* was the dominant species (Fig. S4). In contrast, in pond 4 (without *A. salina*), higher prevalences were recorded in July and August during 2007 and 2008 (S Redón, AJ Green, BB Georgiev, GP Vasileva, F Amat, 2015, unpublished data). Since *A. franciscana* is a poor reservoir (i.e., the cestodes may not circulate effectively in them), adding *A. salina* to the community might increase the prevalence of the infection in the alien host because the added host is a better reservoir (Keesing, Holt & Ostfeld, 2006; Hatcher, Dick & Dunn, 2006). Since *Artemia* are only intermediate hosts, such an explanation would require final avian hosts to be relatively faithful to individual ponds, so that birds that become infected in the pond with *A. salina* are more likely to release cestode eggs there than in other ponds. Another possible and not mutually exclusive explanation for our results is that the final avian hosts are more attracted to ponds holding *A. salina* because this is their preferred prey, leading to greater parasite circulation in these ponds. Owing to weak manipulation by native parasites, *A. franciscana* is much less abundant at the water surface where they are accessible to birds, and as shown in our study, they are less likely to have the red colour preferred by birds (Sánchez et al., 2009b). In turn, this suggests that the generally low infection rates in *A. franciscana* may be accounted for not only by a greater resistance to parasites than native species, but also by a tendency for final hosts (birds) to avoid invaded salt ponds, reducing levels of parasite circulation. A study of how waterbird use differs between salt ponds with native or alien shrimp is required.

### Castration and colour effects in native and alien hosts

In many trophically transmitted parasites, larval parasite stages alter host characteristics such as longevity, size, colour or behaviour to increase the risk of predation by the final host (Moore, 2002; Thomas, Adamo & Moore, 2005; Médoc, Bollache & Beisel, 2006). Parasitic castrators benefit by diverting host resources away from reproduction, allowing faster growth and earlier arrival at the infective stage, or an increase in host lifespan, thus increasing opportunities for transmission (Hurd, Warr & Polwart, 2001). *A. parthenogenetica* infected with *F. liguloides* live longer than uninfected ones (Amat et al., 1991), and this increased host longevity may ensure the complete development of the infective stage from oncosphere to ripe cysticercoid (see Redón et al., 2011), ready to be transmitted to final hosts.

We found significant but mild castrating effects of cestodes in *A. franciscana*, and much stronger effects in native *A. salina*. Despite the smaller sample size, for *A. salina* we could confirm a castrating effect for *A. tringae* and *F. liguloides*. The latter species was already known to have a strong castrating effect on *A. salina* (Amarouayache, Derbal & Kara, 2009) and *A. parthenogenetica* (Amat et al., 1991; Sánchez et al., 2012). In *A. franciscana*, we found significant castration effects for *F. liguloides* and *E. avoceti*, with a stronger effect by the former (a single cysticercoid of *F. liguloides* is able to castrate, whereas two or more are needed for *E. avoceti*). This is the first time that a castration effect has been demonstrated for dilepidid cestodes in *Artemia*, and suggests these effects are widespread and not limited to *F. liguloides*. Our results have important implications for the use of *A. franciscana* in aquaculture, since they show that cestode infections reduce host fecundity, even in the introduced range. This suggests that cyst production in sites within the introduced range where cysts are harvested for aquaculture (e.g., Bohai Bay in China) is likely to be reduced by cestode infections.

This is the first study of the colour-effects of cestodes in *A. franciscana* in the invasive range, and the first to consider the effects in *A. salina* in detail. The association of red colouration with cestode infection is well known for native *A. parthenogenetica*, especially for *F. liguloides*, and appears to be due to parasite manipulation associated with the preference that final hosts have for predating red *Artemia* (Sánchez et al., 2009b; Sánchez et al., 2012). However, it is also possible that this altered pigmentation is caused by a host immune response against infection (e.g., given the antioxidant function and immunostimulating properties of carotenoids) rather than a specific parasitic strategy to ensure transmission. The red colour of cestode-infected *A. parthenogenetica* is partly due to carotenoids (Amat et al., 1991; Sánchez et al., 2006) and carotenoids seem to play an important role in immune defence by providing free radical scavengers against cestode infection (Van der Veen, 2005). Cornet, Biard & Moret (2007) found a positive relationship between carotenoid concentration in haemolyymph and immune parameters (those evolved in the prophenoloxidase cascade) in the crustacean *Gammarus pulex*, suggesting that carotenoids can help to reduce the costs of immunity by limiting self-harming. A positive relationship between carotenoid concentration and the abundance of cestodes (but not of nematodes) was also observed in greylag geese *Anser anser* (Figuerola et al., 2005), suggesting there may be a similar host response to cestode infection across a broad taxonomic range.

The proportion of infected shrimps with a red colour was higher for *A. salina* than for *A. franciscana*, whether considering co-infections or only single infections. This further suggests that the invasive species has greater resistance to cestode infections. Red colouration in *A. salina* was significantly associated with infections by *A. tringae* and *E. avoceti*, and the redness effect of *A. tringae* was stronger than in *A. franciscana*. Surprisingly, we could not detect any effect of *F. liguloides* on *A. salina* colour, suggesting that this native sexual host is more resistent to this parasite than the asexual *A. parthenogenetica*, as predicted by the Red Queen hypothesis. However, red colouration may be of less value to increase transmission to the filter-feeding flamingos that are final hosts of *F. liguloides*, than to shorebirds which are visual predators and final hosts of *E. avoceti* and *Anomotaenia* spp.

Red pigmentation in *A. franciscana* is linked with cestode infection, particularly with dilepidids (*E. avoceti*, *A. tringae*, and *A. microphallos*), and to a lesser extent with flamingo parasites (*F. liguloides*, *F. flamingo* and *G. stammeri*), a finding in line with the higher prevalence and castration effects we recorded for dilepidids. There was a strong positive correlation between red-colour and overall infection-intensity, as previously observed for *A. parthenogenetica* (Sánchez et al., 2006). Ours is the first study to compare redness effects separately in juvenile and adult *Artemia*. For *A. franciscana* juveniles, we found a redness effect for three cestode species, indicating that cestodes manipulate host colour even in early stages of host development. There is a need for detailed research into the nature and function of the pigments causing red colouration in different *Artemia* species and life stages parasitized by different cestodes.

Previous studies in other host–parasite systems have also found native parasites to have less pathological effects in alien hosts than in native hosts (Bauer et al., 2000; Cornet, Sorci & Moret, 2010). For example, in an acantocephalan-gammarid system, Cornet, Sorci & Moret (2010) found that a local parasite *Pomphorhynchus laevis* had less ability to infect and induce pathogenic effects in invasive than in native gammarids.

### Consequences of the invasion of *A. franciscana* for native parasites

Our results demonstrate that *A. franciscana* has accumulated novel parasites following its introduction. However, it is not clear that all the cestodes parasitizing native *Artemia* in the Mediterranean region will be able to persist if all the native populations are replaced by the American species. Coextinctions (i.e., the loss of one especies as a result of the extinction of the species it depends on; Dunn et al., 2009) seem likely in our host–parasite system, especially at a local scale of individual ponds. Cysticercoids have to be able to establish, survive and grow until reaching an infective-stage in brine shrimps, and then reach avian final hosts to complete the parasite’s cycle. It is possible that some cestodes will not be able to infect this new host at a high enough rate or to reach their final hosts with a sufficiently high frequency (according to the very low prevalences and the weaker manipulation observed) to ensure the survival of a viable metapopulation. When introduced species become hosts for native parasites it does not necessarily mean that they play a major role in the life cycles and transmission of these parasites. The reduced levels of infection and reduced castration effects in *A. franciscana* suggest that cestodes may not be able to infect or manipulate the alien host to a sufficient extent to ensure viability. The reduced redness suggests that cestodes are less likely to reach final hosts (through bird predation) when infecting alien *Artemia*, and in turn that the value of salterns for waterbird conservation may be reduced by the invasion. Parasites are an important component of food webs (Lafferty, Dobson & Kuris, 2006; Lafferty et al., 2008) and ecosystem functioning (Hudson, Dobson & Lafferty, 2006), so loss of cestodes may have an impact on the stability of hypersaline ecosystems.

## Conclusions

Cestodes have a greater impact on native *A. salina* than on invasive *A. franciscana* in several ways, suggesting that native cestodes are poorly adapted to these novel hosts. They cause infections of higher prevalence and species richness in the native host. For a given infection intensity, they cause a greater impact on host fecundity, and a greater change in colouration, likely to translate into a higher predation rate by birds. Therefore, cestodes can influence competition between *Artemia* species and are likely to help *A. franciscana* to outcompete native species in the Mediterranean region and other parts of the world. In turn, the viability of these cestode populations across broad scales, where waterbird populations interact with different hypersaline ecosystems, is threatened by the loss of native *Artemia* which may act as a “source” for cestodes whereas *A. franciscana* populations may act as a “sink”.

## Supplemental Information

10.7717/peerj.1073/supp-1 Figure S1 Proportion of juvenile brine shrimps in each sample examined (A) pond CX, (B) pond 4. Proportions are shown for both *Artemia* species when present.

10.7717/peerj.1073/supp-2 Figure S2 Brine shrimp *Artemia* individuals of different colouration Examples highlighted with arrows from left to right are: light-red, dark-red, and not-red.

10.7717/peerj.1073/supp-3 Figure S3 General aspect of adult brine shrimp females with different reproductive status (A) ovigerous, (B) castrated. Females are infected by larval cestodes (indicated by arrows).

10.7717/peerj.1073/supp-4 Figure S4 Temporal dynamics of cestode infection in adult *A. franciscana* from pond CX and proportion of this species in the community, through an annual cycle (January 2009–January 2010) Bars show 95% confidence intervals.

10.7717/peerj.1073/supp-5 Table S1 Comparative data on total cestode prevalence in relation to host community diversity. Data are presented for the native *A. salina* (AS) and the invader *A. franciscana* (AF) when coexisting (syntopic-populations, present study) and from populations without co-ocurrence of other congeneric species or those with small numbers of diploid *A. parthenogenetica* (PD). When many data are available (e.g., for several months, years), the mean value of total prevalence and the range (in parentheses) are given. * mean prevalence of cestode infection was recalculated excluding individuals infected with nematodes recorded in September 2007.

10.7717/peerj.1073/supp-6 Supplemental Information 1 Data for parasite castration effects Raw data used for the analyses of parasite castration effects in adult females of both *Artemia* spp. including all samples available from Ebro delta salterns.

10.7717/peerj.1073/supp-7 Supplemental Information 2 Data for parasite colouration effects Raw data used for the analyses of parasite colour effects in both *Artemia* spp. including all samples available from Ebro delta salterns.

10.7717/peerj.1073/supp-8 Supplemental Information 3 Data from pond CX Raw data for quantitative analyses of infections between alien and native *Artemia* from pond CX during months of coexistence (January to May 2009).

10.7717/peerj.1073/supp-9 Supplemental Information 4 Data *A. franciscana* populations Raw data for comparative infections between *A. franciscana* from syntopic (pond CX) and single (pond 4) populations.

## Additional Information and Declarations

### Field Study Permissions

The following information was supplied relating to field study approvals (i.e., approving body and any reference numbers): This research was conducted under permit from the Ebro Delta Natural Park office provided to FA.
