# Supplementary figures and images for: Comparing cestode infections and their consequences for host fitness in two sexual branchiopods: alien Artemia franciscana and native A. salina from syntopic-populations
Source: PeerJ. 2015 Jul 2;3:e1073. doi: 10.7717/peerj.1073 (PMC4493677; doi:10.7717/peerj.1073)

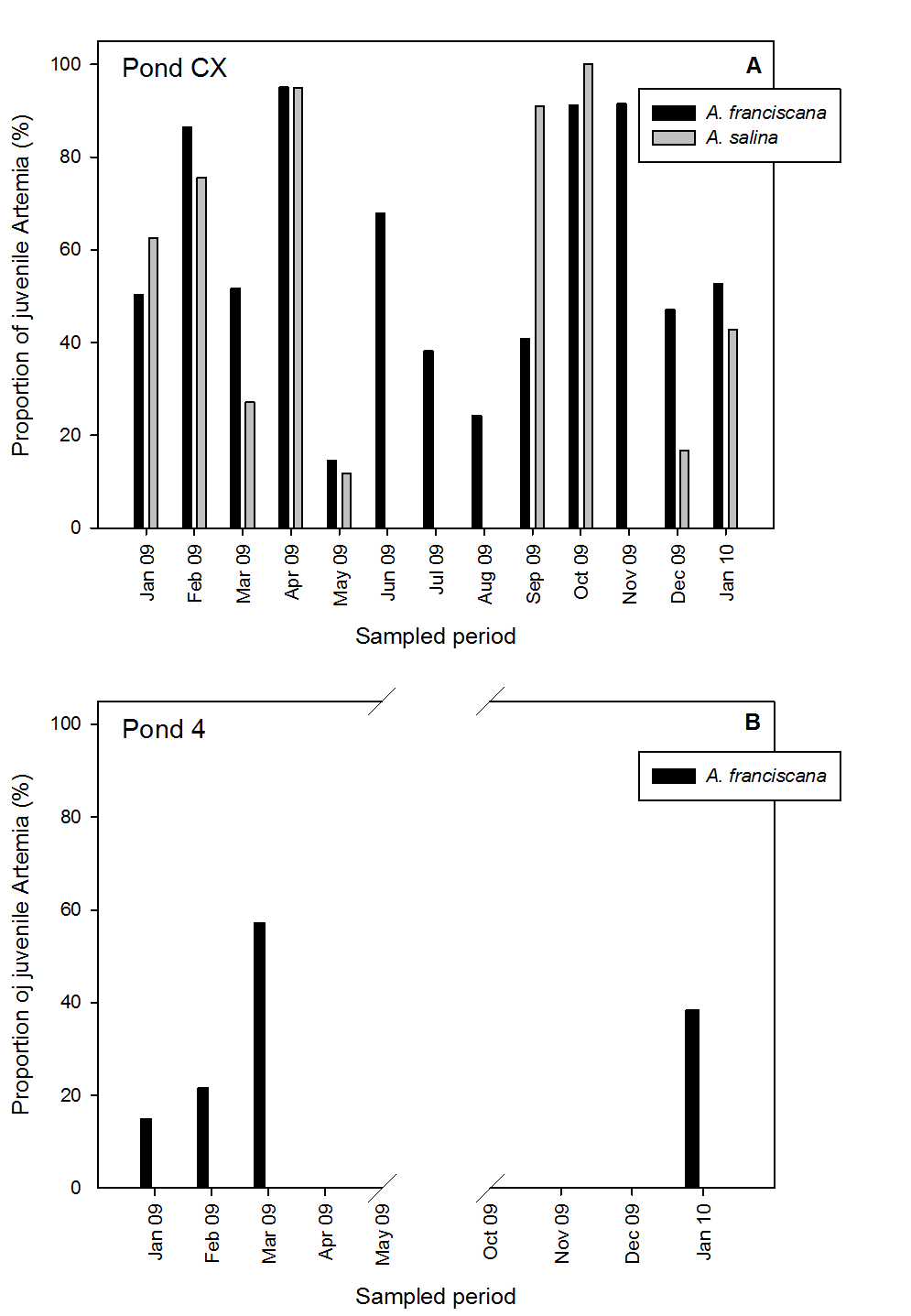

Supplement: Figure S1 — (A) pond CX, (B) pond 4. Proportions are shown for both Artemia species when present. [file peerj-03-1073-s001.png]

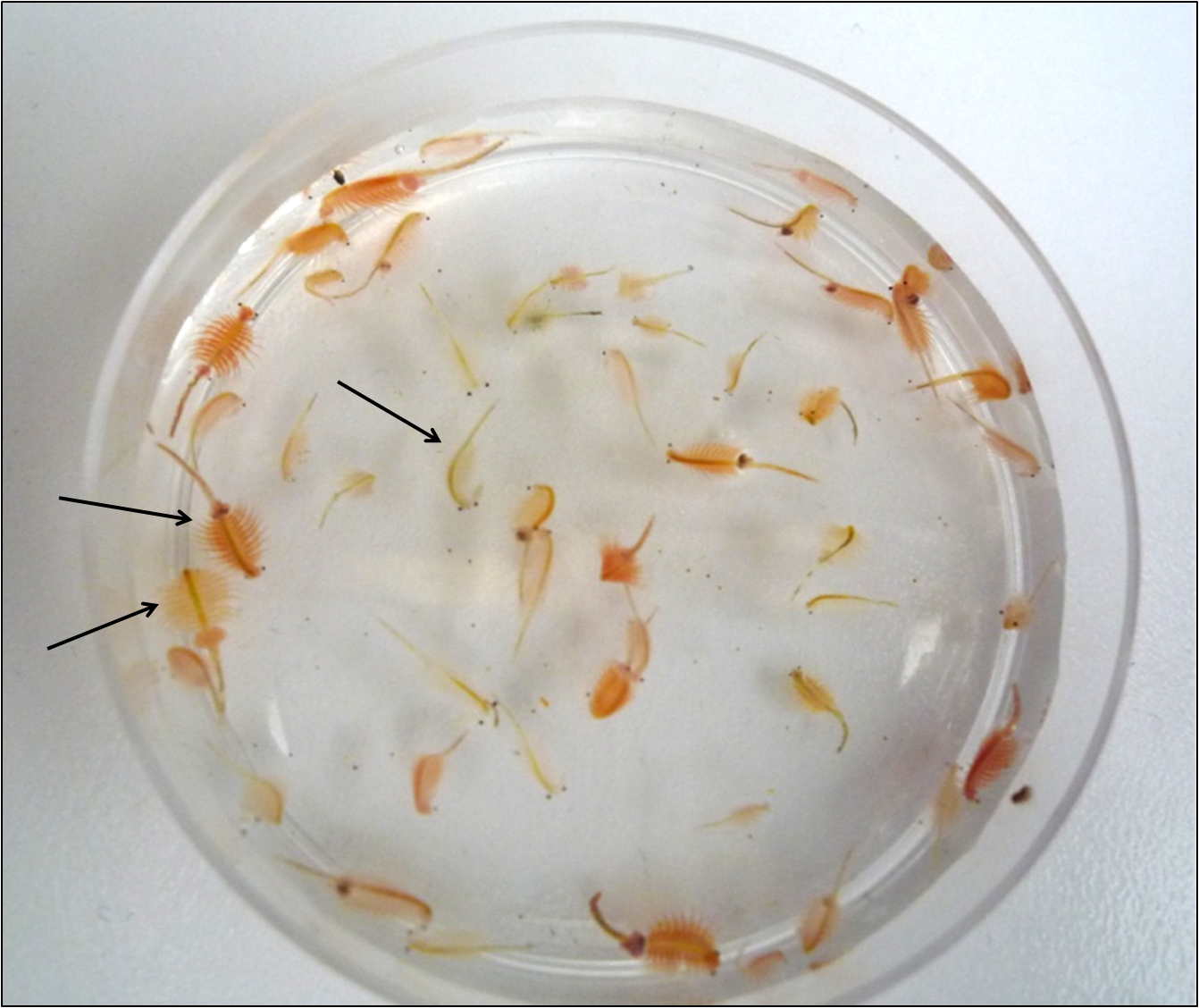

Supplement: Figure S2 — Examples highlighted with arrows from left to right are: light-red, dark-red, and not-red. [file peerj-03-1073-s002.png]

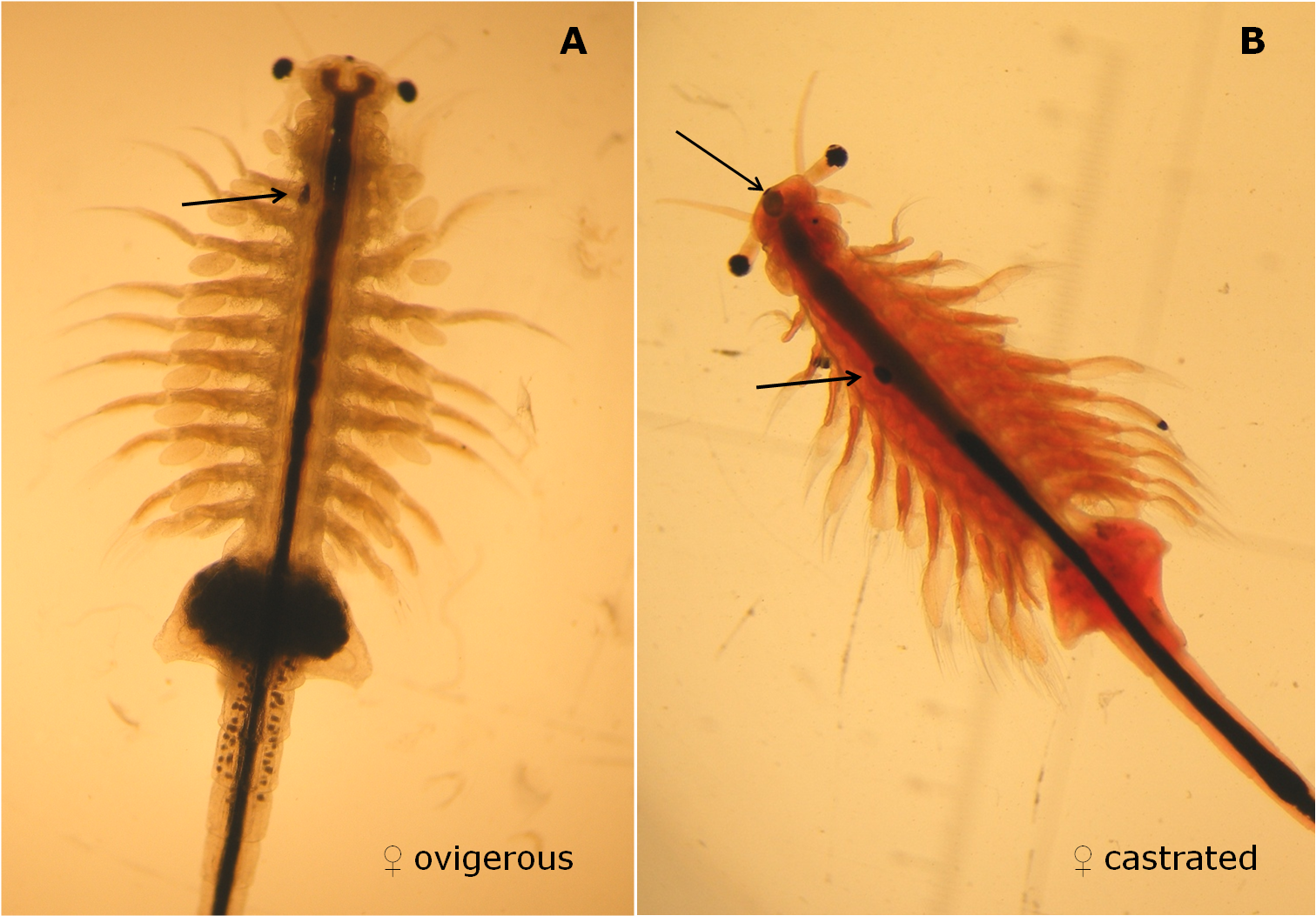

Supplement: Figure S3 — (A) ovigerous, (B) castrated. Females are infected by larval cestodes (indicated by arrows). [file peerj-03-1073-s003.png]

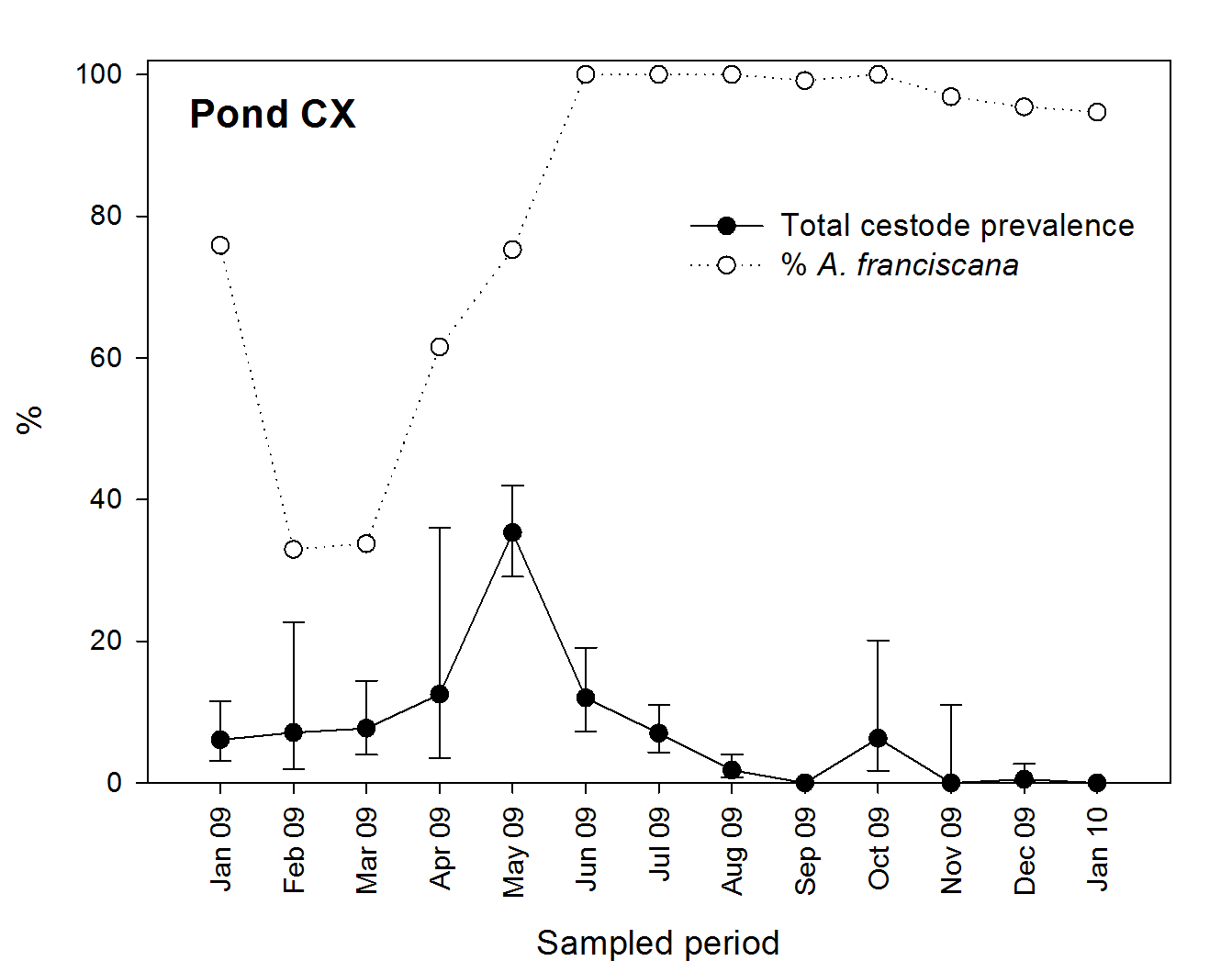

Supplement: Figure S4 — Bars show 95% confidence intervals. [file peerj-03-1073-s004.png]
