# Supplementary material for: Comparing cestode infections and their consequences for host fitness in two sexual branchiopods: alien Artemia franciscana and native A. salina from syntopic-populations
Source: PeerJ. 2015 Jul 2;3:e1073. doi: 10.7717/peerj.1073 (PMC4493677; doi:10.7717/peerj.1073)
Supplement: Table S1 — Data are presented for the native A. salina (AS) and the invader A. franciscana (AF) when coexisting (syntopic-populations, present study) and from populations without co-ocurrence of other congeneric species or those with small numbers of diploid A. parthenogenetica (PD). When many data are available (e.g., for several months, years), the mean value of total prevalence and the range (in parentheses) are given. * mean prevalence of cestode infection was recalculated excluding individuals infected with nematodes recorded in September 2007. [file peerj-03-1073-s005.docx]

| Reference | Location | Total Prevalence (%)  mean (range) | *Artemia* spp. | Sampling date |
| --- | --- | --- | --- | --- |
| Present study | Ebro Delta salterns, Tarragona, Spain | 34.6 (2.4 - 73.5) | AS (+AF) | Jan-May 2009 |
| Georgiev et al., 2007 | Salinas de Cerrillos, Almeria, Spain | 83.0 | AS | September 2005 |
| Sánchez et al., 2013 | Salinas de Cerrillos, Almeria, Spain | 52.1 (27.0 - 72.0) | AS | Oct 2006-Oct 2007 |
| Authors unpublished | San Pedro del Pinatar salterns, Alicante, Spain | 19.3 (4.1 - 48.1) | AS | Feb-June 08, Feb 2009 |
| Authors unpublished | San Pedro del Pinatar salterns, Alicante, Spain | 67.3 (42.2 - 85.7) | AS (< 5% PD) | Feb-June 08, Feb 2009 |
| Authors unpublished | Bras del Port salterns, Alicante, Spain | 3.5 | AS | February 2008 |
| Authors unpublished | La Mata lagoon, Alicante, Spain | 5.7 | AS (< 1% PD) | February 2009 |
|  |  |  |  |  |
| Present study | Ebro Delta salterns, Tarragona, Spain | 13.7 (6.1 - 35.3) | AF (+AS) | Jan-May 2009 |
| Present study | Ebro Delta salterns, Tarragona, Spain | 3.2 (0.5 - 3.0) | AF | Jan-Mar 2009 |
| Authors unpublished | Ebro Delta salterns, Tarragona, Spain | 9.3 (0.0 - 46.4) | AF | Jan-Dec 2008 |
| Georgiev et al., 2007 | Salinas de Santa Bárbara, Cádiz, Spain | 2.0 | AF | August 2005 |
| Georgiev et al., 2007 | Castro Marim Salterns, Algarve, Portugal | 9.0 | AF | August 2005 |
| Georgiev et al., 2007 | Salinas de Las Ánimas, Cádiz, Spain | 24.3 | AF | August 2005 |
| Georgiev et al., 2014 | La Tapa salterns, Cádiz, Spain | 5.5* (0.5 - 13.0) | AF | Dec 2006-Dec 2007 |
|  |  |  |  |  |
| Sánchez et al., 2012 | Aigues Mortes salterns, France | 1.5 | AF (+PD) | July 2008 |
| Sánchez et al., 2012 | Aigues Mortes salterns, France | 0.7 | AF (3% PD) | December 2008 |
